# Complete remission of Merkel cell carcinoma on the upper lip treated with radiation monotherapy and a literature review of Japanese cases

**DOI:** 10.1186/s12957-015-0564-z

**Published:** 2015-04-17

**Authors:** Naoya Kitamura, Riki Tomita, Mayo Yamamoto, Yasumasa Yoshizawa, Eri Sasabe, Tomohiro Yamada, Tetsuya Yamamoto

**Affiliations:** Department of Oral and Maxillofacial Surgery, Kochi Medical School, Kochi University, Kohasu, Oko-cho, Nankoku city, Kochi 783-8505 Japan; Section of Oral and Maxillofacial Surgery, Division of Maxillofacial Diagnostic and Surgical Sciences, Faculty of Dental Science, Kyushu University, Maidashi, Higashi-ku, Fukuoka, 812-8582 Japan

**Keywords:** Merkel cell carcinoma, Radiation monotherapy, Head and neck, Unresectable tumor, Japanese patients

## Abstract

Merkel cell carcinoma is a rare and aggressive neuroendocrine-derived skin cancer arising most commonly on the sun-exposed head and neck skin of elderly and immunocompromised patients. Although a combination of wide excision and adjuvant radiotherapy is the optimal therapeutic approach for Merkel cell carcinoma, radiation monotherapy has recently been recommended for unresectable tumors. We report here a case of Merkel cell carcinoma treated with radiation monotherapy and reviewed Merkel cell carcinoma cases treated with radiotherapy alone in Japan. A 75-year-old man was referred for treatment of a tumor on the upper lip with a swollen submental lymph node. The histopathological diagnosis from biopsied material was Merkel cell carcinoma (T3N1bM0, stage IIIB). The submental lymph node was extirpated and radiation monotherapy was applied according to the 2014 National Comprehensive Cancer Network Guidelines because the Eastern Cooperative Oncology Group Performance Status of the patient was grade 3 and the patient and his family did not desire surgery. The primary site and bilateral upper neck regions were irradiated with 45 Gy followed by 20 Gy irradiation for the primary site alone. Three months after radiotherapy, the tumor seemed to have completely remitted. Approximately 1 year after radiotherapy, no evidence of local recurrence or late metastasis has been noted. Radiation monotherapy should be considered as a curative treatment for Merkel cell carcinoma, particularly in situations where extensive surgery is not favored.

## Background

Merkel cells are afferent sensory receptors that function as mechanoreceptors in the basal layer of the epidermis [1]. Merkel cell carcinoma (MCC) was first described in 1972 by Toker [2] and represents a rare and aggressive neuroendocrine-derived skin cancer that arises most commonly on the sun-exposed head and neck skin of elderly and immunocompromised patients. It has been debated whether MCC arises from malignant transformation of a Merkel cell [3] or from a pluripotent stem cell [4]. This malignancy shows a strong tendency for lymph node metastasis, distant metastasis, and recurrence, which are associated with poor prognosis. The 2014 National Comprehensive Cancer Network Guidelines [5] advocate wide local excision with regional lymph node dissection and adjuvant radiotherapy, but definitive radiation monotherapy has recently been recommended for unresectable tumors, as MCC appears highly sensitive to radiotherapy [6-9]. We present here the case of a patient who achieved complete remission of MCC on the upper lip with neck lymph node metastasis using radiotherapy alone and review MCC cases treated with radiation monotherapy in Japan.

## Case presentation

**Figure 1:**
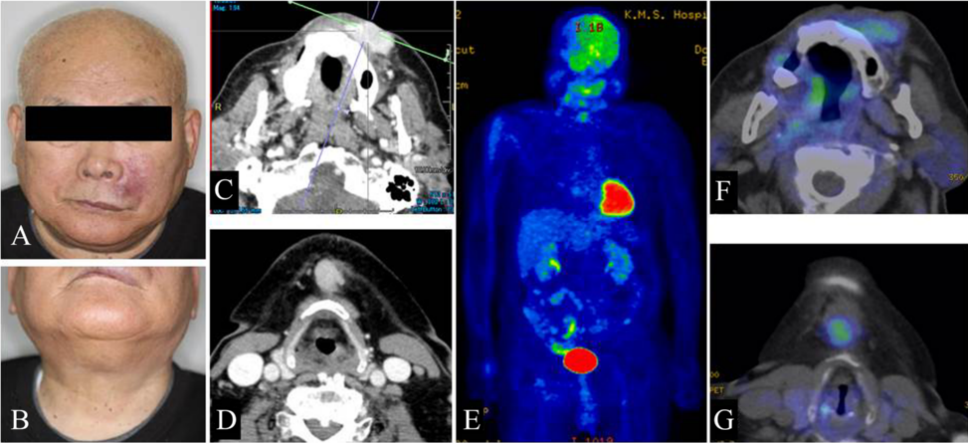
First medical examination. **(A)** Anterior face view. **(B)** Neck view. **(C,D)** Computed tomography of the head and neck region. **(E,F,G)** PET-CT of the whole body and head and neck region.

**Figure 2:**
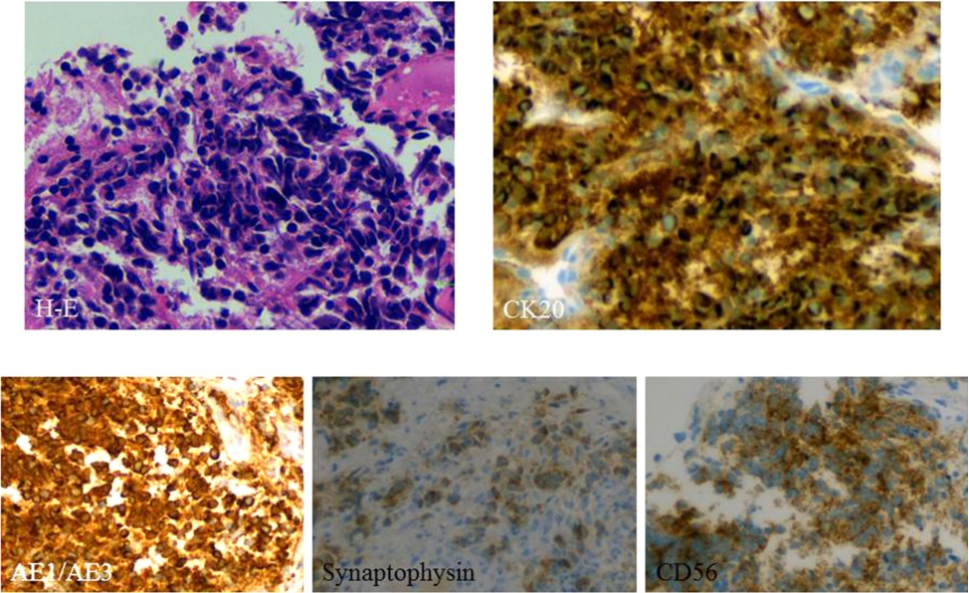
Immunohistochemical staining of metastatic MCC (primary specimen).

**Figure 3:**
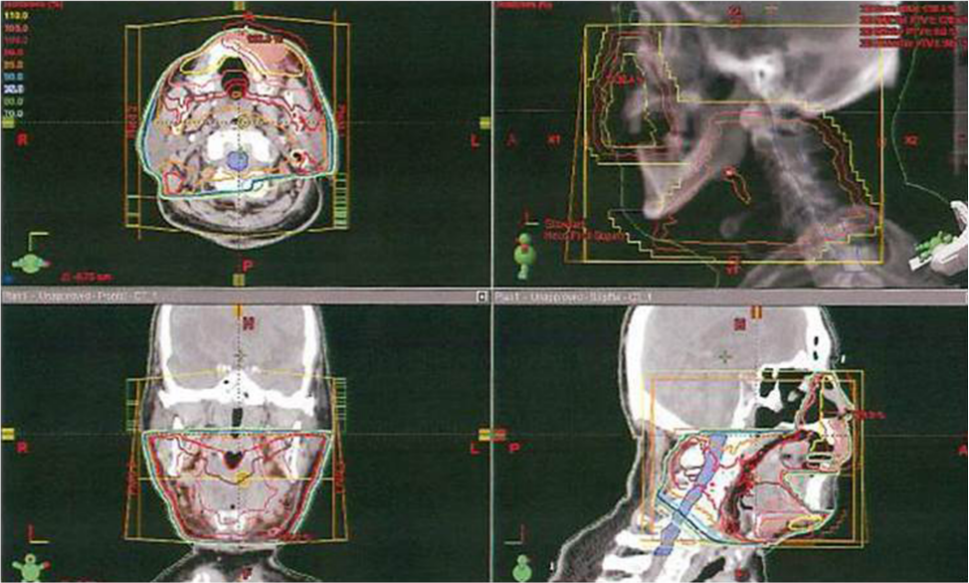
Field settings for radiotherapy. The patient received 4-MV linac X-ray, lateral parallel opposed paired ports for primary site and bilateral levels I and II (45 Gy/25 fr) plus electrons for the primary site alone (20 Gy/10 fr). Field margins were ≥2 cm around the lesion.

**Figure 4:**
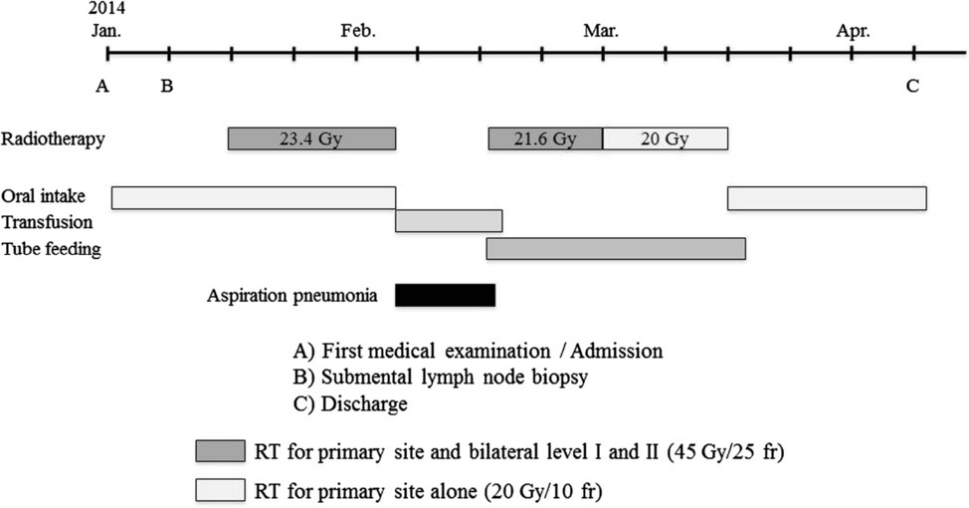
Treatment and course.

**Figure 5:**
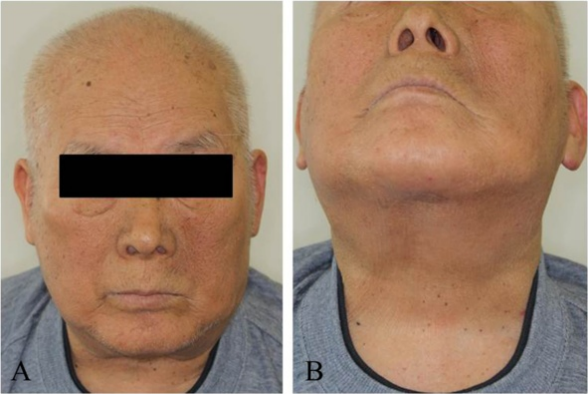
Nine months after radiotherapy. **(A)** Anterior face view. **(B)** Neck view.

A 75-year-old man was referred for treatment of a 5 × 4-cm reddish nodule on the upper lip and a 2 × 2-cm lymph node in the submental region in December 2013 (Figure 1A,B). The medical past history of the patient was prostate cancer, cerebral infarction, and diabetes mellitus. Histopathological examination of the lip tumor revealed an aggregation of atypical tumor cells with scant cytoplasm and immunohistochemical positivity for CK20, AE1/AE3, synaptophysin, and CD58 (Figure 2). The tumor was diagnosed as MCC. Computed tomography indicated approximately 4 × 3-cm and 2 × 2-cm enhanced masses in the upper lip and submental region, respectively (Figure 1C,D). Hot spots of ^18^fluorodeoxyglucose on positron emission tomography were detected at the same sites seen on computed tomography (Figure 1E,F,G). The values of SUVmax in the primary site and submental lymph node were 3.4 and 3.3, respectively. According to the 2014 National Comprehensive Cancer Network Guidelines [5], the submental lymph node was extirpated and the histopathological diagnosis of MCC was confirmed. TNM classification was diagnosed as T3N1bM0, stage IIIB. Treatment with radiation monotherapy was applied because the Eastern Cooperative Oncology Group Performance Status of the patient was grade 3, and the patient and family did not desire surgery. The primary site and bilateral upper neck (levels I and II) regions were irradiated with a dose of 45 Gy/25 fr, followed by irradiation of 20 Gy/10 fr to the primary site alone (Figure 3). Field margins were generally ≥2 cm around the gross tumor volume. During the irradiation period, the planned radiation dose was accomplished using both infusion of electrolyte solution and tubal feeding, although the patient was forced to stop radiation therapy for approximately 10 days due to aspiration pneumonia (Figure 4). At 3 months after radiotherapy, the tumor seemed to show complete remission. As of approximately 1 year after radiotherapy, no evidence of local recurrence or late metastasis has been noted (Figure 5).

## Discussion

MCC is a rare, aggressive, neuroendocrine-derived skin cancer that arises most commonly on the sun-exposed head and neck skin of elderly and immunocompromised patients. MCC at all body sites shows an incidence of around 0.6 per 100,000 capita, but this figure has been increasing over the past 20 years [10]. This malignancy shows a high tendency toward lymph node metastasis, distant metastasis, and recurrence, all of which are predictive of poor prognosis. The acronym “AEIOU” can be applied to describe the clinical features of MCC: asymptomatic/non-tender, expanding rapidly, immune-suppressed, older than 50 years, and ultraviolet-exposed fair skin [11,12]. The 2014 National Comprehensive Cancer Network Guidelines [5] advocate wide local excision (safe margin, 2 ~ 3 cm) with regional lymph node dissection and adjuvant radiotherapy.

On the other hand, for unresectable cases, particularly in patients who would not tolerate wide surgical excision due to comorbidities, or where significant cosmetic or functional deficits would be incurred, definitive radiation monotherapy has recently been recommended, because MCC is highly sensitive to radiotherapy [6-9]. Pape *et al.* found no significant difference in 5-year disease-free and overall survival rates (approximately 90% each) between conventional therapy (surgery followed by radiotherapy) and radiation monotherapy for stage IB to IIB primary 25 MCC patients, respectively [6]. Medina-Franco *et al.* reported that the local relapse rate (LRR) was 10.5% when tumor excision and adjuvant radiotherapy was performed for 1,024 MCC patients [13]. Koh and Veness reported 1-year LRR as 12.5% after performing radiation monotherapy for eight MCC patients [7]. Sundaresan *et al.* reported that 2-year LRR was 15% for 18 MCC patients receiving definitive radiotherapy [8]. Harrington and Kwan studied 179 MCC patients divided into two groups, with 57 receiving radical radiotherapy and 122 receiving radical surgery [9]. No significant differences in 5-year LRR (approximately 10% in each group) and 5-year nodal relapse rate (20% ~ 30% in each group) were seen between the groups.

According to the summary of radiation monotherapy cases reported in Japan, including the present case, that we were able to assemble from the past 20 years, complete response was achieved in 21 of 22 patients (95.5%), and local relapse/late metastasis was seen in 7 of 22 patients (31.8%) (Table 1) [14-30]. Only good cases may have been reported because all Japanese MCC cases were obtained from case reports. Even so, the results suggest that radiation monotherapy in Japan is an effective option, supporting the results of large-scale studies from other countries [6,10-12]. Furthermore, only three cases of radiation monotherapy for Japanese MCC stage III have been reported, including the present case (including two head and neck cases), and the two cases other than this case both showed recurrence (at 2 and 24 months). Careful follow-up will thus be essential in the present case.

**Table 1:** **Merkel cell carcinoma treated with radiotherapy alone in the past 20 years in Japan**

**No.**	**Author**	**Year**	**Age/sex**	**Sites**		**Stage**				**Dose**	**Response**	**Recurrence**	**F/U period**
				**H&N**	**Limbs**	**I**	**II**	**III**	**IV**	**(Gy)**			**(months)**
1	A. Aoki	1995	90F	1					1	40	SD	+	0
2	H. Okada	1995	83F	1			1			30	CR	+	9
3	S. Nasu	1997	88F	1		1				38	CR		14
4	K. Murata	1997	89F	1			1			60	CR		N/A
5	T. Inoue	1999	96M	1			1			50	CR		4
6	Y. Handa	2000	88M		1	1				51	CR		9
7	H. Ono	2001	78F	1		1				50	CR		9
8	S. Seki	2003	60M	1			1			56	CR		21
9			69F	1			1			60	CR		5
10	N. Narita	2004	96M	1			1			66	CR	+	1
11	E. Makino	2005	84F	1		1				48	CR		8
12	T. Nakamura	2005	83F		1			1		60	CR	+	2
13	A. Niiya	2006	72F	1				1		60	CR	+	24
14			66F	1		1				60	CR		18
15	T. Yamakawa	2008	98F	1			1			36	CR		12
16	R. Maeda	2010	86F	1			1			70	CR		19
17	J. Hujitaka	2011	87M	1		1				54	CR		32
18	K. Hanaoka	2011	90F	1			1			28	CR	+	4
19	A. Saito	2012	90F	1			1			45	CR		12
20			80M	1		1				60	CR		22
21			86M		1		1			70	CR	+	12
22	Present case	2014	75M	1				1		65	CR		12
				19	3	7	11	3	1	52.6 ± 12.2	CR:21, SD:1	7/22	11.0 ± 8.4

CR, complete response; F, female; F/U, follow-up; H&N, head and neck; M, male; NA, data not available; SD, stable disease.

Mean age = 83.4 ± 9.9; 8 males, 14 females.

## Conclusions

Radiation monotherapy appears effective for the curative treatment of MCC and should be used, especially in situations where extensive surgery is not favored.

## Consent

Written informed consent was obtained from the patient for publication of this case report and accompanying images. A copy of the written consent is available for review by the Editor-in-Chief of this journal.

## Abbreviations

CR: complete response
F: female
F/U: follow-up
H&N: head and neck
LRR: local relapse rate
M: male
MCC: Merkel cell carcinoma
NA: data not available
SD: stable disease
